# Mapping interictal discharges using intracranial EEG-fMRI to predict postsurgical outcomes

**DOI:** 10.1093/brain/awae148

**Published:** 2024-05-09

**Authors:** William Wilson, Negar Tehrani, Daniel J Pittman, Perry Dykens, Victoria Mosher, Laura Gill, Joseph Peedicail, Antis G George, Craig A Beers, Bradley Goodyear, Pierre LeVan, Paolo Federico

**Affiliations:** Hotchkiss Brain Institute, Cumming School of Medicine, University of Calgary, Calgary, Alberta, T2N 4N1, Canada; Seaman Family MR Research Centre, Foothills Medical Centre, Calgary, Alberta, T2N 2T9, Canada; Hotchkiss Brain Institute, Cumming School of Medicine, University of Calgary, Calgary, Alberta, T2N 4N1, Canada; Seaman Family MR Research Centre, Foothills Medical Centre, Calgary, Alberta, T2N 2T9, Canada; Hotchkiss Brain Institute, Cumming School of Medicine, University of Calgary, Calgary, Alberta, T2N 4N1, Canada; Seaman Family MR Research Centre, Foothills Medical Centre, Calgary, Alberta, T2N 2T9, Canada; Hotchkiss Brain Institute, Cumming School of Medicine, University of Calgary, Calgary, Alberta, T2N 4N1, Canada; Seaman Family MR Research Centre, Foothills Medical Centre, Calgary, Alberta, T2N 2T9, Canada; Hotchkiss Brain Institute, Cumming School of Medicine, University of Calgary, Calgary, Alberta, T2N 4N1, Canada; Seaman Family MR Research Centre, Foothills Medical Centre, Calgary, Alberta, T2N 2T9, Canada; Seaman Family MR Research Centre, Foothills Medical Centre, Calgary, Alberta, T2N 2T9, Canada; Department of Clinical Neurosciences, Cumming School of Medicine, University of Calgary, Calgary, Alberta, T2N 4N1, Canada; Seaman Family MR Research Centre, Foothills Medical Centre, Calgary, Alberta, T2N 2T9, Canada; Department of Clinical Neurosciences, Cumming School of Medicine, University of Calgary, Calgary, Alberta, T2N 4N1, Canada; Hotchkiss Brain Institute, Cumming School of Medicine, University of Calgary, Calgary, Alberta, T2N 4N1, Canada; Seaman Family MR Research Centre, Foothills Medical Centre, Calgary, Alberta, T2N 2T9, Canada; Department of Clinical Neurosciences, Cumming School of Medicine, University of Calgary, Calgary, Alberta, T2N 4N1, Canada; Hotchkiss Brain Institute, Cumming School of Medicine, University of Calgary, Calgary, Alberta, T2N 4N1, Canada; Seaman Family MR Research Centre, Foothills Medical Centre, Calgary, Alberta, T2N 2T9, Canada; Hotchkiss Brain Institute, Cumming School of Medicine, University of Calgary, Calgary, Alberta, T2N 4N1, Canada; Seaman Family MR Research Centre, Foothills Medical Centre, Calgary, Alberta, T2N 2T9, Canada; Department of Radiology, Cumming School of Medicine, University of Calgary, Calgary, Alberta, T2N 4N1Canada; Hotchkiss Brain Institute, Cumming School of Medicine, University of Calgary, Calgary, Alberta, T2N 4N1, Canada; Department of Radiology, Cumming School of Medicine, University of Calgary, Calgary, Alberta, T2N 4N1Canada; Hotchkiss Brain Institute, Cumming School of Medicine, University of Calgary, Calgary, Alberta, T2N 4N1, Canada; Seaman Family MR Research Centre, Foothills Medical Centre, Calgary, Alberta, T2N 2T9, Canada; Department of Clinical Neurosciences, Cumming School of Medicine, University of Calgary, Calgary, Alberta, T2N 4N1, Canada; Department of Radiology, Cumming School of Medicine, University of Calgary, Calgary, Alberta, T2N 4N1Canada

**Keywords:** epilepsy, surgery, electroencephalography, imaging, seizures, markers

## Abstract

Various subjective and objective methods have been proposed to identify which interictal epileptiform discharge (IED)-related EEG-functional MRI (fMRI) results are more likely to delineate seizure-generating tissue in patients with drug-resistant focal epilepsy for the purposes of surgical planning. In this intracranial EEG-fMRI study, we evaluated the utility of these methods to localize clinically relevant regions preoperatively and compared the extent of resection of these areas to postoperative outcome.

Seventy patients admitted for intracranial video-EEG monitoring were recruited for a simultaneous intracranial EEG-fMRI study. For all analyses of blood oxygen level-dependent responses associated with IEDs, an experienced epileptologist identified the most clinically relevant brain activation cluster using available clinical information. The maximum cluster (the cluster with the highest *z*-score) was also identified for all analyses and assigned to one of three confidence levels (low, medium or high) based on the difference of the peak *z*-scores between the maximum and second maximum cluster (the cluster with the second highest peak *z*-value). The distance was measured and compared between the peak voxel of the aforementioned clusters and the electrode contacts where the interictal discharge and seizure onset were recorded. In patients who subsequently underwent epilepsy surgery, the spatial concordance between the aforementioned clusters and the area of resection was determined and compared to postoperative outcome.

We evaluated 106 different IEDs in 70 patients. Both subjective (identification of the clinically relevant cluster) and objective (maximum cluster much more significant than the second maximum cluster) methods of culling non-localizing EEG-fMRI activation maps increased the spatial concordance between these clusters and the corresponding IED or seizure onset zone contacts. However, only the objective methods of identifying medium and high confidence maps resulted in a significant association between resection of the peak voxel of the maximum cluster and postoperative outcome. Resection of this area was associated with good postoperative outcomes but was not sufficient for seizure freedom. On the other hand, we found that failure to resect the medium and high confidence maximum clusters was associated with a poor post-surgical outcome (negative predictive value = 1.0, sensitivity = 1.0).

Methods to identify higher confidence EEG-fMRI results are needed to localize areas necessary for good postoperative outcomes. However, resection of the peak voxel within higher confidence maximum clusters is not sufficient for good outcomes. Conversely, failure to resect the peak voxel in these clusters is associated with a poor post-surgical outcome.

## Teaser

Wilson *et al.* evaluate the utility of intracranial EEG-fMRI as a tool to help predict outcomes in epilepsy surgery. Removal of the brain area where peak activation (as revealed by fMRI) is associated with brief seizure discharges is necessary, but not sufficient, for good post-surgical outcomes.

## Introduction

Simultaneous scalp EEG and functional MRI (EEG-fMRI) has been used in research since the 1990s to better understand and delineate areas critical for seizure generation. For example, the maximum blood oxygen level-dependent (BOLD) response to interictal epileptiform discharges (IEDs) has been found to localize the spike onset zone, a potential marker of the epileptogenic zone in patients undergoing pre-surgical evaluation.^1,2^ Despite this, EEG-fMRI has undergone little clinical adoption.^1,3^

Early retrospective work evaluating postoperative outcomes using the BOLD response to IEDs on a small patient cohort showed the sensitivity and specificity of this preoperative marker of the epileptogenic zone to be 87.5% and 76.9%, respectively.^4^ The authors further demonstrated that if brain tissue that co-localized with the maximal BOLD change was removed at surgery, then the positive predictive value of a good postsurgical outcome was 70%, and if the BOLD changes resided outside the resection area, the negative predictive value was 90.9%.^4^ This finding supported the potential use of EEG-fMRI clinically; however, several barriers to widespread use have persisted, including 10%–15% of scalp EEG-fMRI analyses yielding no significant result, difficulty interpreting diffuse activation maps and handling multiple IED types and locations within the same patient. Furthermore, a more recent study involving a larger patient cohort, showed more modest performance metrics (sensitivity 59%, specificity 61%, positive predictive value 47% and negative predictive value 72%).^5^ The authors observed poor outcomes when the peak BOLD response was not resected, however, the removal of the peak BOLD response itself was not sufficient to yield seizure freedom.^5^

Several objective approaches have been proposed to further refine the analysis and interpretation of IED-related BOLD responses.^5-7^ These approaches focused on providing criteria to cull BOLD activation maps that may mislead clinical decision-making and on identifying value-added maps, typically at the expense of reducing the yield of EEG-fMRI investigations overall. One such approach defined high confidence maps as those where the magnitude of the maximum cluster (peak *z*-score) was much greater than the next highest cluster (second peak *z*-score), which resulted in superior localization of regions, such as the seizure onset zone (SOZ).^6^ Importantly, the majority of these approaches were carried out using scalp EEG-fMRI. The use of intracranial electrodes has advantages, such as greater sensitivity to IEDs generated in deeper structures and avoidance of signal attenuation by scalp and skull.^8-10^ Whether the implementation of intracranial EEG-fMRI provides an increased yield of these high confidence IED-related BOLD activation maps has not yet been demonstrated.

We have previously explored the identification of a single most clinically relevant cluster to localize the spike onset zone associated with IEDs.^7^ We proposed that a subjective assessment of all significant BOLD activation, not only the maximum, would better define a cluster commensurate with IED origin and potentially with seizure generation. We found that, particularly in the analysis of IEDs originating from extra-temporal structures, the clinically relevant cluster was closer to the location of the electrode where the local field potential was observed than when using the maximum cluster alone.^7^ The utility of the clinically relevant cluster to predict postoperative outcome has not been assessed, however.

In the present study, we performed intracranial EEG-fMRI to assess and compare the ability of both subjective and objective methods of refining IED-related BOLD maps to predict post-surgical outcome. We further sought to determine whether IED-related BOLD maps obtained using intracranial EEG-fMRI would result in a greater yield of high confidence clusters as compared to scalp EEG-fMRI.

## Materials and methods

### Participants

Seventy subjects undergoing intracranial video-EEG monitoring (VEM) were recruited between May 2008 and April 2021. The inclusion criteria were as follows: (i) >18 years old; (ii) capable of providing informed consent; (iii) no MRI contraindications; and (iv) a diagnosis of focal epilepsy. Subjects were recruited prior to discharge, at which point therapeutic doses of anti-seizure medications were resumed. The study was approved by the Conjoint Health Research Ethics Board of the University of Calgary.

### Data acquisition

#### Intracranial EEG

Each subject’s intracranial VEM data were reviewed by an experienced epileptologist (P.F., J.S.P., L.G.) to select up to eight electrodes to be used during the iEEG-fMRI acquisition. These electrodes were connected to either a two-tailed (up to 20 channels, Subjects ICE001–007) or an eight-tailed MRI-compatible electrode connector (up to 64 channels, Subjects ICE008–070; Compumedics Neuroscan) and routed to a SynAmps RT headbox and amplifier system (Compumedics Neuroscan). EEG data were acquired at 10 or 20 kHz using Scan 4.4 (prior to 2020) or Curry8 (2020 to present) software (Compumedics Neuroscan). For Subjects ICE025–070, the EEG system clock was synchronized to the MRI scanner clock. Real-time artefact rejection and filtering were used to facilitate monitoring of the EEG throughout the acquisition.

#### MRI

Subjects ICE001–007 were scanned using a 3 T GE Signa LX whole body scanner with a receive-only eight-channel phased-array head-receive/body transmit coil, and Subjects ICE008–070 were scanned using a 3 T GE Discovery MR750 whole body scanner with an eight-channel receive-only phased-array head coil (GE Healthcare). Subjects were closely monitored during the acquisition.

The MRI protocol included multislice anatomical imaging [spoiled gradient-recalled echo two-dimensional (2D) multislice sequence: echo time = 2.1 ms, repetition time = 150 ms, flip angle = 18°, 128 × 128 matrix, 24 slices 5.00 mm thick], anatomical 3D T_1_-weighted imaging (echo time = 3.8 ms, repetition time = 9.3 ms, flip angle = 12°, 24-cm field of view, 320 × 256 × 64 matrix, 0.47 × 0.47 × 2.00 mm slices) and fMRI (gradient-recalled echo planar imaging: echo time = 30 ms, repetition time = 1500 ms, flip angle = 65°, 24-cm field of view, 64 × 64 matrix, 24, 25, or 29 slices 3.75 × 3.75 × 5.00 mm).

All subjects were encouraged to sleep to increase the probability of IED occurrence.^11^ Subjects were scanned for up to 60 min (comprising 800 volumes) in multiple runs no longer than 20 min each.

### Data preprocessing

#### Intracranial EEG

EEG data postprocessing was performed using an in-house MATLAB algorithm (MathWorks, MATLAB 2021b). This algorithm performed the following steps: (i) re-referencing of each channel using a within-electrode common average; (ii) 0.5–500 Hz bandpass filter; (iii) average artefact subtraction^12^; (iv) principal component analysis where components were removed in order of variance explained until the frequency spectrum of the cleaned signal was most similar to a baseline comparator recorded outside the scanner; and (v) downsampling to 2500 Hz.

Each dataset was then reviewed by two experienced electroencephalographers (two of Y.A., P.F., J.S.P., S.S., L.G., C.B.J., K.M.K., A.S.) to identify the timing and location of IEDs. Specifically, IED location was identified as the electrode contacts where phase reversal (bipolar montage) and maximum amplitude (referential montage) were observed. IEDs arising from distinct locations in the same patient were grouped separately, herein referred to as distinct IED types. Any disagreements between the two reviewers were reviewed and resolved by both reviewers.

#### Functional MRI

Functional MRI analyses were performed using the FMRIB Software Library (FSL, http://www.fmrib.ox.ac.uk/fsl). Preprocessing of the functional MRI data included slice timing correction, spatial smoothing using a Gaussian kernel with a full-width at half-maximum of 6 mm, high-pass temporal filtering (Gaussian-weighted least-squares straight line fitting, with sigma = 50.0 s) and non-brain removal using the Brain Extraction Tool (BET).^13^ Motion correction was carried out using Motion Correction: FMRIB Linear Image Registration Tool (MCFLIRT).^14^ For motion over 1.5 mm, the corresponding frames were removed and the scan was split into two. If splitting the scan resulted in a continuous segment shorter than five continuous minutes, it was excluded from analysis. FSL’s multivariate exploratory linear optimized decomposition into independent components (MELODIC) tool was used to decompose each functional scan into 60 components. All components were visually reviewed by two reviewers (W.W., V.M.) to identify signal components attributed primarily to noise or artefact,^15^ which were then regressed from the data. Disagreement between the two reviewers were reviewed and resolved by both reviewers. Functional MRI data were registered to the low resolution and high resolution anatomical images using FMRIB linear image registration tool (FLIRT).^14,16^

### Event-related analysis

Similar to previous scalp EEG-fMRI studies, four different haemodynamic response functions (HRFs) with varying times to peak at 3, 5, 7 and 9 s, were used to convolve with the timings of IEDs, resulting in four regressors being modelled for each IED.^17^ Statistical analyses were carried out using FSL Expert Analysis Tool (FEAT) implementing FMRIB Improved Linear Model (FILM), with local autocorrection applied for each functional scan. FMRIB Local Analysis of Mixed Effect (FLAME) was then used to create a statistical average from each subject’s scans, employing a fixed effects model (i.e. random effects variance was forced to zero). Z-maps were corrected using a cluster threshold of *z* = 3.1 with a cluster *P* threshold of 0.01. Finally, at each voxel location, the most significant *z*-value from the four HRFs was selected to create a composite *z*-map. Composite *z*-maps were then registered to the subject’s high resolution structural image using FLIRT.

### Cluster identification and refinement

For each IED analysis, only the positive BOLD responses were considered, as we have previously reported negative BOLD responses to be less informative with a more poorly understood mechanism.^1,2,18^ Within each positive BOLD *z*-map, we identified (i) the cluster with the highest *z*-value, the maximum BOLD cluster (Max); (ii) the cluster with the second highest *z*-value, the second maximum BOLD cluster (Max’); and (iii) the cluster in which the peak voxel was closest to the electrode contacts where the IEDs were observed, the closest cluster (Closest). One additional cluster, the clinically relevant cluster (CR), was identified, as previously described.^7^ Briefly, an epileptologist experienced with fMRI (P.F.) examined the entire BOLD activation map for all IEDs and selected one cluster as the most ‘relevant’ cluster. This was done subjectively in two steps: (i) the presumed locations of the IED origin and SOZ were identified from the patient’s available clinical data (history, VEM data, SPECT, PET, etc.) by consensus of the Calgary Comprehensive Epilepsy Program; followed by (ii) visual examination of the features (*z*-value and shape) of the cluster nearest to these locations, as well as the Max cluster, to discard clusters more likely to represent spurious activations (e.g. small volume, low *z*-value or crosses anatomical boundaries) in favour of larger, higher *z*-value clusters located near the involved electrode contacts. Notably, the reviewer could choose to select no cluster if there was no convincing activation.

Maximum clusters, but not the other cluster types, were then classified into three levels of confidence (low, medium and high). The low confidence group was defined as any maximum cluster that survived correction for multiple comparisons using a cluster threshold of *z* = 3.1 with a cluster *P* threshold of 0.01. For the medium confidence group, we implemented an inequality previously described as identifying *z*-maps where the difference between the magnitudes of the *z*-value of the maximum and second maximum clusters is sufficiently large as to indicate a higher probability of corresponding to the SOZ given by:


$$|z_{1}|\;x\;0.0813\;+\;(\left|z_{1}|-|z_{2}\right|)\;x\;0.6135\;>\;1^{6}$$

Here, z_1_ refers to the peak *z*-value of the maximum cluster, and z_2_ refers to the peak *z*-value of the second maximum cluster. For the high confidence group, we implemented a second more stringent inequality that identifies maximum clusters with an even greater difference between the maximum and second maximum *z*-values given by:


$$|z_{1}|\;x\;0.025\;+\;(\left|z_{1}|-|z_{2}\right|)\;x\;0.080\;>\;0.302^{5}$$

### Electrode to BOLD distances

For each cluster type (Max, Max’, Closest and CR) the distance was measured from the peak voxel (X_1_, Y_1_, Z_1_) to the locations of the electrode contacts where the maximum amplitude of the corresponding IEDs were observed (X_2_, Y_2_, Z_2_) using:


$$Distance_{12}=\sqrt{{\left(X_{2}-\;X_{1}\right)}^{2}+{\left(Y_{2}-\;Y_{1}\right)}^{2}+{\left(Z_{2}-\;Z_{1}\right)}^{2}}$$

In instances where more than one electrode contact was involved, their geometric centre was calculated using the 3D coordinates of each electrode contact using the general equation:


$$X_{\mathrm{GC}}\;=\;(X_{1}\;+\;X_{2}\;+\;\ldots X_{n})/n$$

For distances between each of the cluster types to the presumed SOZ, IEDs were paired with SOZs ipsilateral to the IED. The distance was then measured from the peak voxel of each of these clusters to the electrode contacts involved in the clinically defined SOZ using the same procedure as above.

### Comparison to area of resection

All subjects that underwent epilepsy surgery underwent postoperative structural imaging to identify the resection area. Binary masks of the volume of resection were manually created using FSLeyes.^19^ Postoperative images were then co-registered to the preoperative structural image in two stages using the DRAMMS (deformable registration via attribute matching and mutual-saliency weighting) toolbox^20^: (i) a linear affine registration of the target and source images; followed by (ii) a constrained non-linear deformation, implementing a weighted mask of the region indicated by the resection mask. The effectiveness of the transformations was verified visually, to ensure satisfactory overlap of major landmarks without excessive warping. The preoperative Z-map from each IED analysis was then overlaid onto the postoperative image and classified into one of four groups as in previous studies^4,5^: (i) fully concordant (FC) if the peak voxel of the maximum BOLD cluster was inside the area of resection; (ii) partially concordant (PC) if the peak voxel of the maximum cluster was outside the area of resection but within 2 cm of the margin and part of the maximum cluster overlapped with the area of resection; (iii) partially discordant (PD) if no part of the maximum BOLD cluster was resected, but another, less significant cluster was included in the area of resection; and (iv) fully discordant (FD) if no significant cluster was included in the area of resection.

### Surgical outcome

Seizure freedom was assessed >12 months after the procedure using the Engel scale. Good outcome was defined as Engel class I (free of disabling seizures) and class II (rare disabling seizures) and poor outcome was defined as Engel class III (worthwhile improvement) and class IV (no worthwhile improvement).^21^ The Engel scale was chosen over the International League Against Epilepsy (ILAE) surgical epilepsy scale to allow for direct comparison to previous EEG-fMRI reports of postoperative outcomes. Moreover, the Engel scale has been shown to be highly correlated with the ILAE scale with similar inter-rater reliability.^22^

#### Classification of evidence

With respect to assessing the relationship between resection of the maximum cluster and postoperative outcome, this study is rated class IV because of its retrospective design.

#### Statistical analyses

All statistical analyses were performed using Prism version 9.3.1 (GraphPad, La Jolla, CA). The distribution of the data was visualized and tested for normality using the Shapiro-Wilk test before additional statistical testing. Considering that cluster volumes and distances to electrodes are strictly positive, their distributions were found to be skewed and therefore did not follow a normal distribution. To carry out hypothesis testing we performed Kruskal-Wallis one-way analysis of variance on continuous variables between groups followed by Dunn’s tests for *post hoc* pair-wise comparisons. We used binary classification for prediction of postoperative outcome, comparing good versus poor outcomes between either fully concordant versus fully discordant (FC versus FD) results, or fully concordant and partially concordant versus fully discordant and partially discordant (FC + PC versus FD + PD) results, all using Fisher’s exact test. Significance thresholds were set to *P* < 0.05. Values are expressed as mean with standard deviation (SD).

## Results

### Subject characteristics

Intracranial EEG-fMRI was generally well tolerated, with all 70 subjects undergoing at least 10 min of functional image acquisition without discomfort. Ten subjects were excluded due to poor data quality as follows: inability to satisfactorily remove gradient switching artefact from EEG (*n* = 5), <5 mins of continuous functional data remained after our motion correction procedure (*n* = 3), and no convincing IEDs seen (*n* = 2). Table 1 shows a summary of subject demographic, clinical and event data. Of the remaining 60 subjects, 29 were female (48%). The mean age at the time of study was 35.8 years old (range = 20.6–64.7 years), with a mean duration of epilepsy of 18.2 years (range = 2.9–53.2 years). Seizure frequency ranged from daily to yearly: 22/60 subjects (37%) had daily seizures, 12/60 subjects (20%) had weekly seizures, 24/60 subjects (40%) had monthly seizures, and 2/60 subjects (3%) had yearly seizures. Twenty-four of 60 subjects (40%) had a potentially epileptogenic lesion noted on MRI: hippocampal sclerosis (8/60, 13%), focal cortical dysplasia (5/60, 8%), other malformations of cortical developments (4/60, 7%) and other lesions (7/60, 12%). Thirty-eight patients subsequently underwent resective surgery, targeting the temporal lobe in 21/38 subjects (55%), the frontal lobe in 7/38 subjects (18%) and multiple lobes in 10/38 subjects (26%). No surgeries targeted the parietal or occipital lobes exclusively.

**Table 1:** Summary demographic, clinical and event data

	Total (*n* = 60)
**Age**	–
Mean (SD)	35.8 (12.0)
Range	20.6–64.7
**Sex, *n* (%)**	–
Female	29 (48%)
Male	31 (52%)
**Handedness, *n* (%)**	–
Right	50 (83%)
Left	5 (8.5%)
Ambidextrous	5 (8.5%)
**Age at onset**	–
Mean (SD)	17.6 (11.5)
Range	0.0–57.0
**Duration of epilepsy**	–
Mean (SD)	18.2 (12.8)
Range	2.9–53.2
**Epilepsy (hemisphere), *n* (%)**	–
Right	21 (35%)
Left	24 (40%)
Bilateral	15 (25%)
**Epilepsy (lobe), *n* (%)**	–
Temporal	34 (57%)
Frontal	12 (20%)
Parietal	0 (0%)
Occipital	0 (0%)
Multiple lobes	14 (23%)
**Seizure frequency, *n* (%)**	–
Daily	22 (37%)
Weekly	12 (20%)
Monthly	24 (40%)
Yearly	2 (3%)
**Lesion^a^, *n* (%)**	–
Hippocampal sclerosis	8 (13%)
Focal cortical dysplasia	5 (8%)
Other malformation of cortical development	4 (7%)
Other	7 (12%)
**Number of IED groups/subject**	–
Mean (SD)	2.0 (0.9)
Range	1–5

IED = interictal epileptiform discharge; SD = standard deviation.

^a^Subjects may have more than one lesion noted.

### Clinically relevant cluster

**Figure 1:**
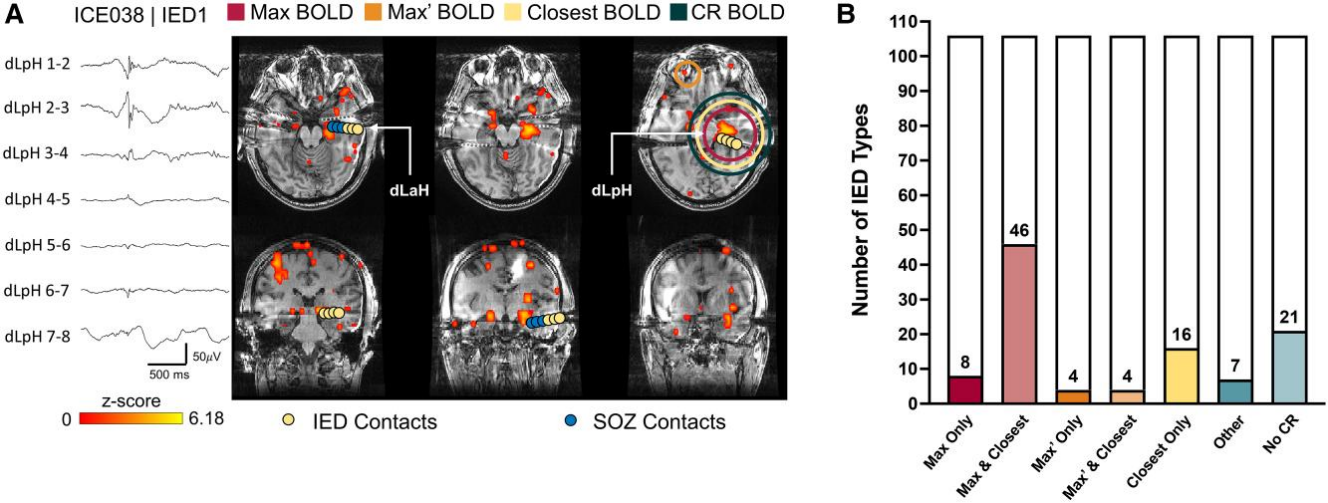
**Selection of the clinically relevant BOLD response from all available clusters**. (**A**) Example of a blood oxygen level-dependent (BOLD) activation map associated with left posterior hippocampal interictal epileptiform discharges. Yellow and blue dots indicate the contacts where interictal discharges or seizure onset was observed, respectively. In this example, the Max BOLD cluster (red circle) is also the Closest BOLD cluster (yellow circle) to the interictal epileptiform discharge (IED) contacts and was selected as the clinically relevant (CR) BOLD cluster (dark green circle). The Max’ BOLD cluster (orange circle) was observed contralateral to the discharge and extends superiorly. (**B**) Composition of the clinically relevant BOLD cluster for all 106 IED types studied. Note, a single cluster may be identified as both the Max or Max’ cluster and Closest cluster (denoted Max and Closest cluster or Max’ or Closest cluster, respectively) or belong to only one group (denoted Max Only, Max’ Only or Closest Only). SOZ = seizure onset zone.

A total of 117 different IED types were analysed from 60 subjects, which yielded 106 maps with at least one significant positive BOLD cluster. Figure 1 provides an example of the BOLD response to an IED (Fig. 1A) as well as the distribution of cluster types selected as the most clinically relevant (Fig. 1B). A clinically relevant cluster was identified in 85/106 (80%) IED analyses. In 66/85 selections (78%), the Closest cluster was chosen as most clinically relevant, where the Closest cluster was also the Max cluster in 46/66 selections (70%), also the Max’ cluster in 4/66 selections (6%) and neither the Max nor Max’ in 16/66 selections (24%). In 8/85 selections (9%), the Max but not the Closest cluster was chosen, in 4/85 selections (5%) the Max’ but not the Closest cluster was chosen, and in 7/85 selections (8%), a cluster other than the Max, Max’ or Closest clusters was chosen.

### Interictal epileptiform discharge analyses

A total of 117 IED types were identified (mean number of IED types per subject = 2.0, range = 1–5). Nineteen subjects had one type, 28 subjects had two types, eight subjects had three types, three subjects had four types and one subject had five types. The number of IEDs recorded during acquisition ranged from 5 to 3389 (mean = 479.3, SD = 621.9). The analysis of these 117 IED types yielded 106 maps with at least one significant positive BOLD cluster. Supplementary Fig. 1A and B shows the *z*-scores and cluster volumes for the different cluster types. The mean *z*-scores for the Max, Max’, Closest and CR clusters were 6.54 (SD = 2.38), 5.06 (SD = 0.95), 5.83 (SD = 2.54) and 6.63 (SD = 2.52), respectively and the corresponding cluster volumes were 40.0 cm^3^ (SD = 113.4), 6.6 cm^3^ (SD = 22.5), 20.2 cm^3^ (SD = 81.9) and 42.8 cm^3^ (SD = 121.1). CR clusters had significantly higher *z*-scores and larger cluster volumes than both the Max’ and Closest clusters [*z*-score: H(3) = 26.92, *P* < 0.001, Dunn’s adjusted, CR versus second Max: *P* = 0.0008, CR versus Closest: *P* = 0.0083; cluster volume: H(3) = 16.79, *P* = 0.0008, Dunn’s adjusted, CR versus second Max: *P* = 0.0044, CR versus Closest: *P* = 0.0120]. There was no difference between the mean *z*-scores or cluster volumes of the Max and CR clusters.

**Figure 2:**
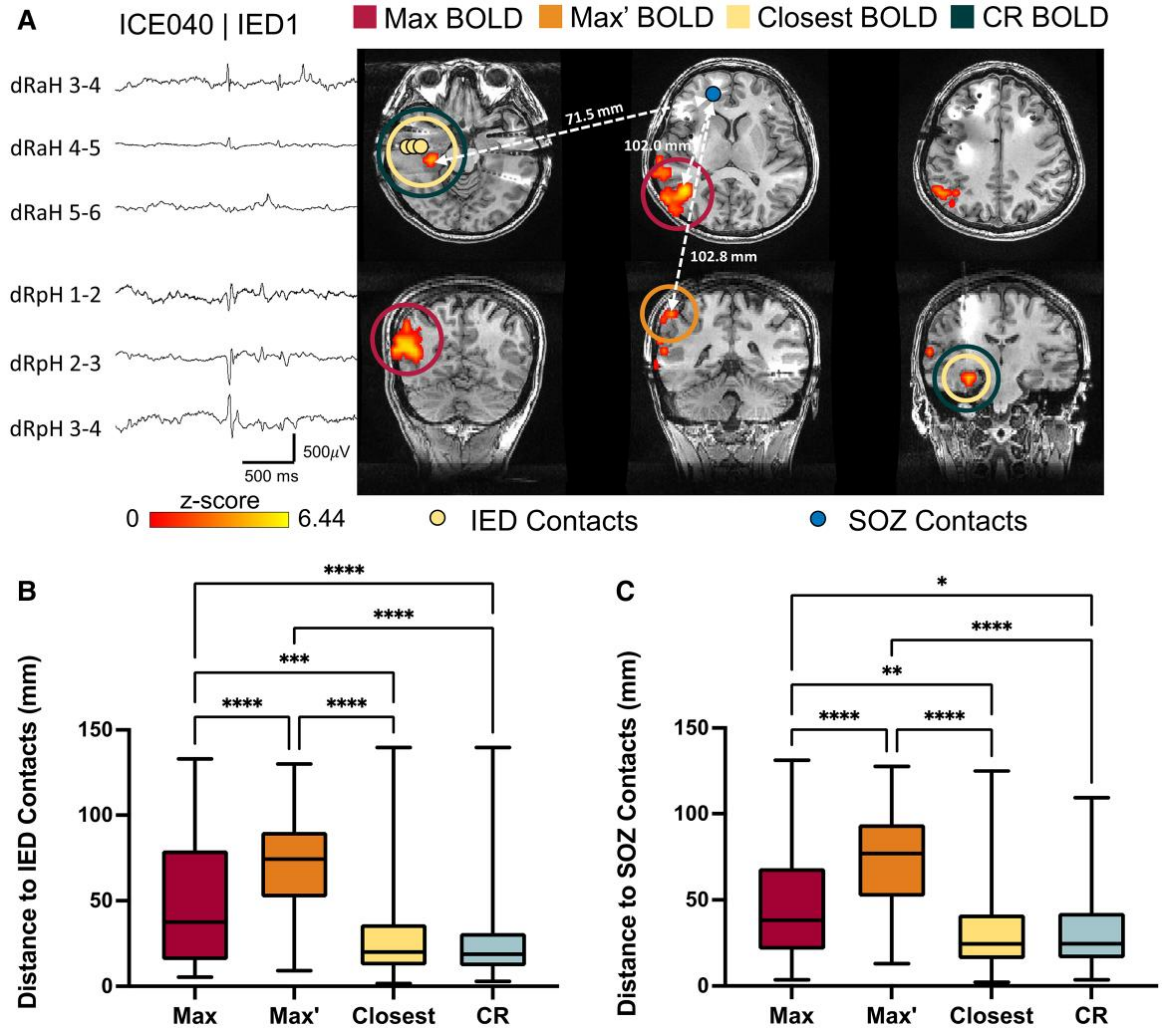
**Distances between the BOLD clusters of interest and electrode contacts**. (**A**) Example of a blood oxygen level-dependent (BOLD) activation map associated with interictal epileptiform discharges (IEDs) recorded from the right anterior and posterior hippocampus. Yellow and blue dots indicate the contacts where interictal discharges or seizure onset was seen, respectively. In this example, the Closest BOLD cluster (yellow circle) to the contacts where IEDs were seen, and not the Max BOLD cluster (red circle), was selected as the clinically relevant (CR) BOLD cluster (dark green circle). The Max’ BOLD cluster (orange circle) was located in more superior slices. Distances (indicated by white hatched arrows) were measured between the peak voxel of each BOLD cluster of interest and both the IED contacts as well as seizure onset zone (SOZ) contacts (71.5 mm to the Closest BOLD cluster, 102.0 mm to the Max BOLD cluster and 102.8 mm to the Max’ BOLD cluster). (**B**) Mean distance between the BOLD cluster of interest and the associated electrode contacts where IEDs were recorded. (**C**) Mean distance between the BOLD cluster of interest and paired (ipsilateral) SOZ contacts. *P < 0.05, **P < 0.01, ***P < 0.001, ****P < 0.0001. Error bars represent standard mean error.

Figure 2A shows the BOLD activation map for Subject ICE040 associated with a right hippocampal discharge (location indicated by yellow dots). The distances from the different cluster types and the IED and SOZ contacts are also shown.

As shown in Fig. 2B, the average distance from the peak voxel of the Max, Max’, Closest and CR clusters to the corresponding most active IED contacts for all 106 IED types identified was 49.4 mm (SD = 35.8), 71.0 mm (SD = 28.3), 31.1 mm (SD = 29.6) and 27.5 mm (SD = 25.1), respectively and 45.8 mm (SD = 30.5), 72.1 mm (SD = 28.9), 31.3 mm (SD = 23.7) and 30.9 mm (SD = 21.5) to the SOZ contacts, respectively. Distances to the IED and SOZ contacts were significantly different between all groups except for the CR and Closest clusters [IED: H(3) = 97.83, *P* < 0.001, SOZ: H(3) = 89.31, *P* < 0.001, Dunn’s adjusted *P* < 0.05; Fig. 2C]. The Max’ clusters had the largest distances, while Max clusters had shorter distances. Overall, the Closest and CR clusters were the closest to both IED and SOZ contacts.

**Figure 3:**
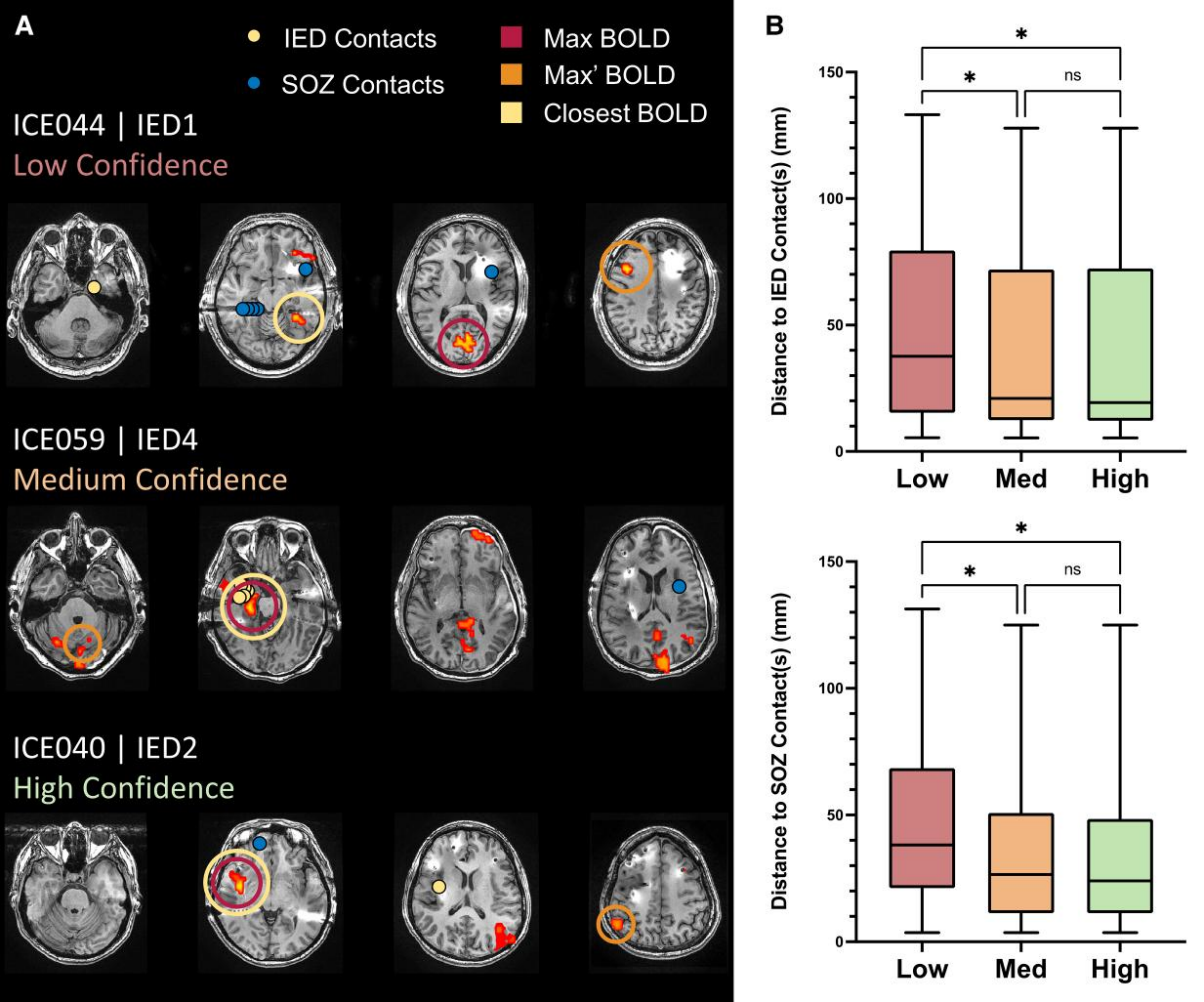
**Classification of maximum cluster confidence**. (**A**) *Top*, *middle* and *bottom rows* show examples of low, medium and high confidence interictal epileptiform discharge (IED)-associated blood oxygen level-dependent (BOLD) activation maps, respectively. Yellow and blue dots indicate the contacts where interictal discharges or seizure onset was seen, respectively. The Maximum and Closest BOLD clusters are indicated by a surrounding dark red circle or yellow circle, respectively. (**B**) Mean distances from the peak voxel of the IED-associated Maximum BOLD clusters (low, medium or high confidence) to the IED (*top*) and seizure onset zone (SOZ) contacts (*bottom*). Note that higher confidence clusters are necessarily also included in the group mean calculation for the lower confidence groups. That is, the medium confidence group is a subset of the low confidence group, and the high confidence group is a subset of both the medium and low confidence groups. Significant differences are indicated by asterisks, ns = non-significant differences. Error bars represent standard error.

These analyses were repeated using the Max BOLD clusters stratified by confidence level (Fig. 3). Examples of low, medium and high confidence IED-related BOLD activation maps are shown in Fig. 3A. Overall, we found that higher confidence maps, unsurprisingly, had a larger mean *z*-score [H(3) = 13.67, *P* = 0.0011; low: 6.54 versus med: 7.76 versus high: 8.09] accompanied by a larger cluster volume [H(3) = 8.39, *P* = 0.0151; low: 40.0 cm^3^ versus med: 69.8 cm^3^ versus high: 74.7 cm^3^; Supplementary Fig. 1C and D]. The low confidence group was 49.4 mm (SD = 35.8) and 45.8 mm (SD = 30.5) from the IED and SOZ contacts, respectively. The medium confidence group was 36.6 mm (SD = 33.1) and 35.2 mm (SD = 27.6) from the IED and SOZ contacts, respectively, and the high confidence group was 36.3 mm (SD = 34.3) and 33.2 mm (SD = 27.9) from the IED and SOZ contacts, respectively. As shown in Fig. 3B, the distances from medium and high confidence Max BOLD clusters to both the IED and SOZ contacts were significantly shorter than the low confidence group [IED: H(3) = 8.90, *P* = 0.0117, SOZ: H(3) = 7.24, *P* = 0.0268, Dunn adjusted *P* < 0.05], but no additional reduction in distance between the medium and high confidence groups was observed.

### Surgical outcome and concordance

**Figure 4:**
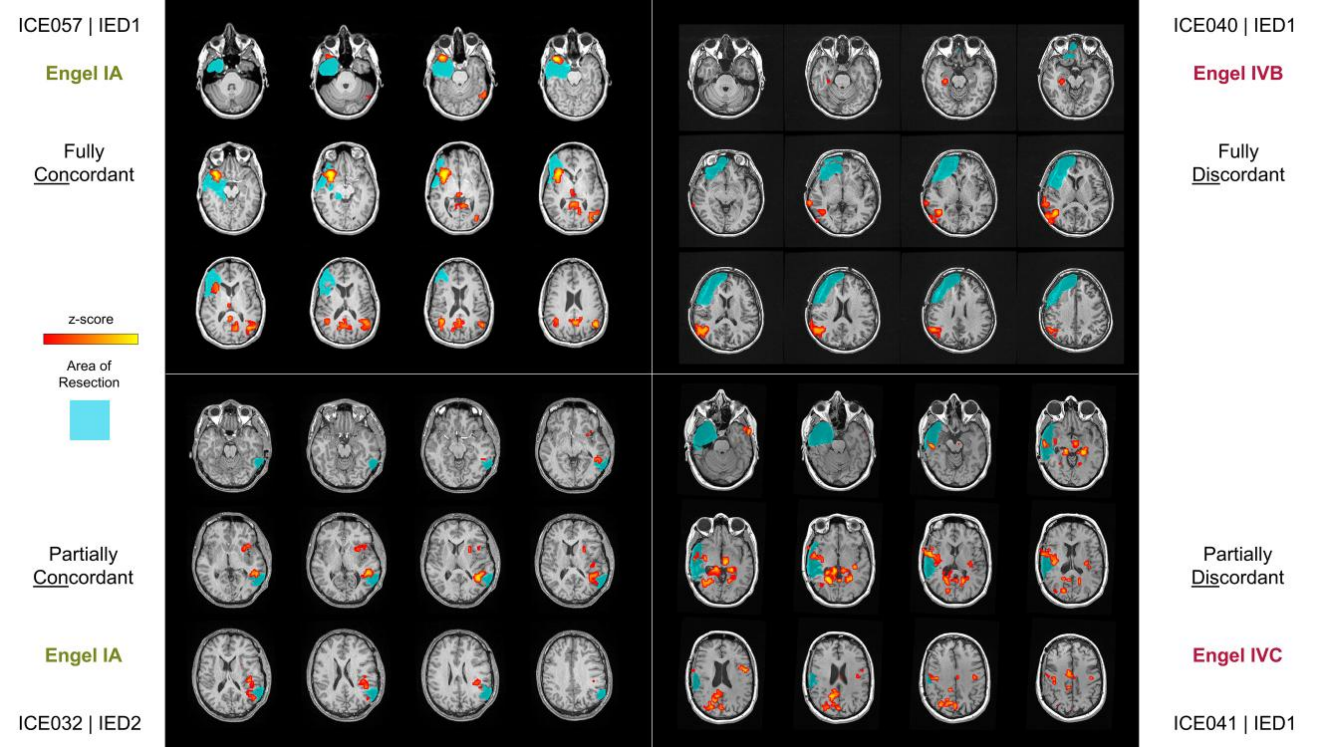
**Classification of spatial concordance between the Max BOLD cluster and the area of resection**. *Top left*: An example of a subject where the Max blood oxygen level-dependent (BOLD) cluster was fully concordant with the area of resection (i.e. peak voxel was resected) and the subject is seizure-free (Engel IA). *Top right*: An example of a subject where the Max BOLD cluster was fully discordant with the area of resection (i.e. no cluster was resected) and the subject continues to experience seizures (Engel IVB). *Bottom left*: An example of a subject where the Max BOLD cluster was partially concordant with the area of resection (i.e. peak voxel not resected, but overlap with Max BOLD cluster) and the subject is seizure-free (Engel IA). *Bottom right:* An example of a subject where the Max BOLD cluster is partially discordant with the area of resection (i.e. no part of the Max BOLD cluster was resected, but another less significant cluster was resected) and the subject continues to experience seizures (Engel IVC).

Thirty-eight of 60 patients underwent surgery, accounting for 68 maps with at least one significant positive BOLD cluster. A good outcome (Engel I and II) was achieved in 23/38 (61%) subjects. To evaluate surgical outcome as it relates to the surgical removal of the Max cluster, we created two binary classifications of either fully concordant versus fully discordant (FC versus FD) or fully concordant and partially concordant versus fully discordant and partially discordant (FC + PC versus FD + PD) to generate two × two confusion matrices for each confidence level (Table 2). Figure 4 shows examples of each classification type (FC, PC, PD, FD). The medium and high confidence groups were significant at a 5% level for non-random association (*P* = 0.0124 and *P* = 0.040, respectively, Fisher’s exact test) for the FC versus FD comparison, but not the FC + PC versus FD + PD comparison (*P* = 0.263 and *P* = 0.500, respectively, Fisher’s exact test). The low confidence group did not reach statistical significance for either the FC versus FD or FC + PC versus FD + PD comparisons. Similarly, we found that both the medium and high confidence groups had no instances of false negatives. That is, there were no instances where the subject had a good outcome and the peak voxel of the Max cluster was not resected. This has the consequence of a negative predictive value and sensitivity of 1.0 in both cases. In contrast, several false-positives were observed. That is, the peak voxel of the Max cluster was resected, but the patient had a poor postoperative outcome. This is consistent with a previous finding that resection of this area is necessary but not sufficient for a good postoperative outcome.^5^ We also examined the performance of resection of the CR cluster as it relates to postoperative outcome and found no significant associations.

**Table 2:** Contingency table and performance statistics for different confidence levels

	Max BOLD cluster									Clinically relevant BOLD cluster								
	Low confidence level			Medium confidence level			High confidence level			Low confidence level			Medium confidence level			High confidence level		
	Engel outcome			Engel outcome			Engel outcome			Engel outcome			Engel outcome			Engel outcome		
	I–II	III–IV	All	I–II	III–IV	All	I–II	III–IV	All	I–II	III–IV	All	I-II	III-IV	All	I-II	III-IV	All
***z*-map**																		
Fully concordant	12	6	18	**6**	**6**	**12**	**5**	**6**	**11**	13	8	21	7	6	13	6	6	12
Fully discordant	6	10	16	**0**	**10**	**10**	**0**	**8**	**8**	1	4	5	0	4	4	0	3	3
All	18	16	34	**6**	**16**	**22**	**5**	**14**	**19**	14	12	26	7	10	17	6	9	15
**Performance**																		
*P-*value	0.087	–	–	0.0124*	**–**	–	**0**.040*	**–**	**–**	0.117	–	–	0.088	–	–	0.185	–	–
OR (95% CI)	3.33	0.78–13.89		**21**.**00**	**1.01–438.25^a^**		**14**.**38**	**0.67–309.86^a^**		6.50	0.73–84.51		10.38	0.47–231.64^a^		7.00	0.30–164.40^a^	
Sensitivity (95% CI)	0.67	0.44–0.84		**1**.**00**	**0.61–1.00**		**1**.**00**	**0.57–1.00^a^**		0.93	0.69–1.00		1.00	0.65–1.00		1.00	0.61–1.00	
Specificity (95% CI)	0.63	0.39–0.82		**0**.**63**	**0.39–0.82**		**0**.**57**	**0.33–0.79**		0.33	0.14–0.61		0.40	0.17–0.69		0.33	0.12–0.65	
PPV (95% CI)	0.67	0.44–0.84		**0**.**50**	**0.25–0.75**		**0**.**45**	**0.21–0.72**		0.62	0.41–0.79		0.54	0.29–0.77		0.50	0.25–0.75	
NPV (95% CI)	0.63	0.39–0.82		**1**.**00**	**0.72–1.00**		**1**.**00**	**0.68–1.00**		0.80	0.38–0.99		1.00	0.51–1.00		1.00	0.44–1.00	
***z*-map**																		
Concordant + partially concordant	16	10	26	9	8	17	7	8	15	21	11	32	12	8	20	9	8	17
Discordant + partially discordant	22	20	42	7	12	19	6	9	15	11	12	23	3	6	9	2	4	6
All	38	30	68	16	20	36	13	17	30	32	23	55	15	14	29	11	12	23
**Performance**																		
*P-*value	0.314	–	–	0.263	–	–	0.500	–	–	0.149	–	–	0.177	–	–	0.365	–	–
OR (95% CI)	1.46	0.53–3.76		1.93	0.50–6.44		1.31	0.34–5.43		2.08	0.71–6.60		3.00	0.58–12.98		2.25	0.36–13.83	–
Sensitivity (95% CI)	0.42	0.28–0.58		0.56	0.33–0.77		0.54	0.29–0.77		0.66	0.48–0.80		0.80	0.55–0.93		0.82	0.52–0.97	
Specificity (95% CI)	0.67	0.49–0.81		0.60	0.39–0.78		0.53	0.31–0.74		0.52	0.33–0.71		0.43	0.21–0.67		0.33	0.14–0.61	
PPV (95% CI)	0.62	0.43–0.78		0.53	0.31–0.74		0.47	0.25–0.70		0.66	0.48–0.80		0.60	0.39–0.78		0.53	0.31–0.74	
NPV (95% CI)	0.48	0.33–0.62		0.63	0.41–0.81		0.60	0.36–0.80		0.52	0.33–0.71		0.67	0.35–0.88		0.67	0.30–0.94	

Values in bold indicate groups with statistically significant associations. BOLD = blood oxygen level-dependent; CI = confidence interval; NPV = negative predictive value; PPV = positive predictive value; OR = odds ratio.

Fisher's exact test was used to calculate *P-*values.

^a^For the high confidence group, the OR was calculated by adding 0.5 to all values.

## Discussion

We performed an intracranial EEG-fMRI study of 70 subjects aimed at evaluating subjective and objective measures of refining IED-related BOLD responses as markers of the spike and seizure onset zones as well as predictors of post-surgical outcome. We found that application of either subjective (CR cluster) or objective (graded confidence for the Max cluster) methods improved localization of the spike and seizure onset zones. However, only the objective methods of identifying medium and high confidence maps resulted in a significant positive association between resection of the peak voxel of the Max cluster and postoperative outcome.

### Using cluster refinement methods to improve localization

For a clinical investigation to prove useful, determining the conditions under which the results are relevant and reliable must be known. Significant efforts have been made to determine in which situations EEG-fMRI results are useful and for which they are not. Resultant BOLD activation maps may be disregarded entirely if the clusters are overly diffuse or are commensurate with patterns of resting state networks.^5,7^ For subjects with more than one distinct IED type, multiple studies have suggested selecting for the IED map with the most significant clusters (highest *t* or *z*-value),^5^ the most clinically relevant clusters,^7^ or to indeed consider them all. For IED-related maps with several discrete clusters, additional criteria may be used to select the most salient for surgical planning. Most notably, the cluster with the maximum BOLD response is often used and is suggested to define the spike onset zone.^23^

Importantly, it is increasingly recognized that it may be necessary to create additional criteria to account for cases when the maximum BOLD response may be spurious or misleading. Multiple approaches have been suggested, primarily involving the use of a subjectively identified CR cluster^7^ or the use of confidence criteria.^5,6^ With the implementation of selection criteria, EEG-fMRI has increasing potential for use during the pre-surgical work-up in patients with drug-resistant epilepsy. We employed the same confidence criteria as previously reported in scalp EEG-fMRI studies to determine if this method would similarly identify clusters more relevant to surgical decision-making in an intracranial EEG-fMRI paradigm.^5,6^ The congruence of our results with these other studies, which likewise demonstrated very high negative predictive values, supports adoption of this approach in future clinical and research applications of EEG-fMRI to improve post-surgical outcomes. The use of these criteria comes at the expense of greatly reduced yield, however. We found that the application of criteria to select for only medium and high confidence maps decreased the available maps to 36/68 (53%) and 30/68 (44%), respectively, and that 55/68 (81%) maps were determined to have a CR BOLD. This is slightly more than values reported in a scalp EEG-fMRI study that showed that 32/106 (30%) IEDs included in their analyses could be included in the high confidence group.^5^ Therefore, intracranial EEG-fMRI may confer a greater yield of both IEDs detected and significant BOLD activation compared to scalp EEG-fMRI. Importantly, the indications for intracranial versus scalp EEG-fMRI should be considered the same as the indications for intracranial versus scalp video-EEG monitoring, including when the location of the origin of epileptogenic activity is unclear or it is suspected to arise from deep structures.

### The maximum cluster: necessary but not sufficient

Perhaps the most relevant question that remains is why EEG-fMRI does not provide meaningful positive predictive value (PPV). Applying the principle of neurovascular coupling, one would expect the haemodynamic response to neuronal activity related to IEDs to be within a reasonable distance of the EEG. Indeed, we found the mean distance from the peak voxel to the corresponding IED contacts was 49.4 mm and 45.8 mm to the SOZ contacts. However, given that removal of the clinically determined SOZ using existing clinical investigations results in seizure freedom in <60% of cases, proximity to this imperfect marker of the true target, the epileptogenic zone, may be a limitation. Further, in 3/6 analyses associated with a poor postoperative outcome despite resection of the peak voxel of the medium or high confidence Max clusters (false positive), the subject had a separate IED type that resulted in a Max cluster that was not resected (true negative). Therefore, in some cases, surgical failure may be a result of multiple foci rather than incomplete removal of the full extent of a single focus.

The presence of more than one IED type presents a challenge to interpreting a patient’s EEG-fMRI findings, especially if they point to different surgical targets. The use of confidence criteria may help facilitate patient-level interpretation in these instances in two key ways. First, this process will eliminate IED types that generate lower confidence ‘noise’ clusters related to poor data quality or IEDs less associated with the epileptogenic zone. Indeed, we found that 26/40 patients (65%) with more than one IED type had no more than one IED type that satisfied medium or high confidence fMRI criteria. Second, if more than one high confidence IED type is identified, sufficient validation of these clusters has been performed to reasonably consider multi-focal epilepsy in these patients, which has a significant impact on the procedure offered and the probability of postoperative success. Taken together, all IED types that produce high confidence clusters should be considered in surgical planning.

### Partial concordance/discordance: a grey area

In this study we found that only the comparison between fully concordant and fully discordant groups resulted in a significant association with postoperative outcome. This is contrary to previous reports that have found a significant association when analyses included both full and partial concordance or discordance.^5^ This suggests that the peak voxel is necessary to resect, while attempting to make a prediction based on any part of the *z*-map, including even other regions within the Max cluster, is unreliable. This is not altogether surprising given the lack of consensus in the field of fMRI on how to adequately control for false positive voxels while preserving sufficient sensitivity to find an effect. That is, how to threshold statistical maps in fMRI remains an unanswered question. The extent of a cluster is often very sensitive to the correction method implemented and therefore evaluating the partial resection of regions of lesser significance is likely equally sensitive to correction method.

It was previously reported in a retrospective scalp EEG-fMRI study, that in the high confidence group, no case of a good outcome was observed if the peak voxel was >2 cm from the resection cavity, even if another less significant cluster was removed (partially discordant). This suggested that it is the Max cluster alone (peak voxel and cluster extent) that identifies the area necessary to remove. In the current study, in the high confidence group, we similarly never observed a good outcome if the peak voxel was >2 cm from the resection cavity and no cluster whatsoever was removed (fully discordant). However, we did find several cases where the peak voxel was >2 cm from the resection area, but another less significant cluster was removed (partially discordant) and the patient had a good outcome. A potential explanation for the different findings could be that the peak voxel alone does not necessarily need to be resected, but that 2 cm may be too narrow of a margin of error. A major advantage of using intracranial rather than scalp EEG in the implementation of EEG-fMRI is that intracranial electrodes provide better spatial resolution and can more precisely identify the source of electrical activity. However, susceptibility artefact observed in fMRI images, surrounding the platinum-iridium macroelectrodes commonly used, introduces spatial distortions and signal loss. We have previously found that the distance from an electrode contact needed to recover 70% of the T_2_* signal of grey matter is 1.75 cm,^24^ effectively attenuating the signal and reducing the temporal signal-to-noise ratio within that radius and therefore our ability to detect signal associated with our model. Given this, the Max cluster could be broken up into a series of smaller clusters of variable significance when intersecting one or more electrodes. Therefore, when we observed good outcomes with the removal of any non-Max clusters, this may represent the removal of an important cluster whose significance was marred by proximity to an electrode. Accordingly, for intracranial EEG-fMRI, modifying the partial concordance criteria to a larger distance may result in similar findings as previous studies employing scalp EEG-fMRI.

### Utility of the clinically relevant cluster

The CR cluster was proposed as a means of providing flexibility when selecting a cluster that may represent the origin of IED generation, given the variety of patterns often observed in EEG-fMRI analyses (e.g. diffuse, bilateral, etc.). Most notably, the CR cluster may be of greatest use when there is extensive susceptibility artefact due to intracranial electrodes that may consequently limit the sensitivity to the spike onset zone, and/or when the Max cluster is observed in a non-meaningful location for the purpose of surgical planning (e.g. thalamus, brain stem, resting network node). We found that the CR cluster most often represents the cluster closest to both the electrode contacts where the local field potentials of the IEDs are recorded, as well as contacts that comprise the SOZ. This has important practical implications given the SOZ itself is an imperfect marker of the epileptogenic zone, providing sufficient spatial information for seizure freedom in <60% of cases.^25,26^ That is, while the CR cluster is a very good marker of the SOZ, it may not provide novel spatial information required to inform surgical planning beyond that provided by the ictal EEG alone. We observed this as a non-significant association in our postoperative analysis. The Max cluster, especially those that pass additional confidence criteria, in contrast, appears to provide an objective marker that we found necessary to remove to result in postoperative seizure freedom.

### Limitations

The use of iEEG-fMRI allows direct sampling from deep brain structures, such as the hippocampus, which can yield a sufficient number of IEDs varying in amplitude and duration of discharge for analysis. However, several limitations exist with this technique. A limitation in our study is gradient-induced signal dropout surrounding the depth electrodes. The use of a 3 T scanner has been shown to yield a dropout of 40%–70% within ∼1.75 cm of electrodes.^24^ Although this is a limitation, studies have demonstrated IED-related BOLD changes to be found in the immediate area surrounding the most active contacts where IEDs are observed on EEG.^27-29^ Moreover, one group identified BOLD activation immediately surrounding depth electrodes and found the duration of the IED and the amplitude of the BOLD signal in these voxels were correlated.^30^ Together, this suggests that although there is significant signal loss, sufficient lower amplitude fluctuations persist as to permit for correlation with the timing of IEDs. Given these technical considerations, the selection of only one cluster, the Max cluster, may prove to be a limitation, however this must be balanced with the advantages of an objective, more readily interpretable approach to these results.

In general, no significant activation was seen in ∼10% of all patients undergoing intracranial or scalp EEG-fMRI. The use of confidence criteria applied to maps with significant activation identifies between 50% (using intracranial EEG) and 30% (using scalp EEG) datasets for subsequent clinical interpretation. Given the resource-intensive nature of EEG-fMRI, future work is needed to determine if the remaining low confidence maps truly cannot be used (e.g. due to technical failure) or if these results are useful in identifying patients who are poor candidates for surgery (e.g. presence of diffuse networks).

In the present study, the CR cluster was subjectively selected by an experienced epileptologist from all available clusters as the one that best represents the location of the spike onset zone, using all available clinical information including medical history, seizure monitoring data and imaging from other modalities.^7^ This method of evaluation of EEG-fMRI results has the potential to improve localization, especially when the Max cluster is distant to the location of IEDs seen on EEG. However, the inter-rater reliability of selecting the CR cluster has not been assessed and therefore generalizability across centres may be limited.

## Conclusion

In this study, we provide intracranial EEG-fMRI evidence that corroborates similar findings from scalp EEG-fMRI studies. That is, only Max IED-related BOLD clusters that have been further screened for their significance relative to other clusters within the same activation map (i.e. higher confidence maps) should be used in surgical planning. Furthermore, we found that resection of the peak voxel is paramount for surgical success as resections that remove portions of the Max cluster without removing the peak voxel proper are more likely to result in surgical failure.

## Supplementary Material

awae148_Supplementary_Data

## Data Availability

Anonymized data that support the findings of this study are available upon request from the corresponding author and approval by the Conjoint Health Research Ethics Board of the University of Calgary. The data are not publicly available as they contain patient identifying information that could compromise the privacy of research participants.

## Appendix 1

The Calgary Comprehensive Epilepsy Program collaborators are: Yahya Aghakhani, Walter Hader, Nathalie Jetté, Colin Josephson, Karl Martin Klein, William Murphy, Neelan Pillay, Andrea Salmon, Shaily Singh, Yves Starreveld, Samuel Wiebe.
