# Supplementary material for: Mapping interictal discharges using intracranial EEG-fMRI to predict postsurgical outcomes
Source: Brain. 2024 May 9;147(12):4157–68. doi: 10.1093/brain/awae148 (PMC11729745; doi:10.1093/brain/awae148)
Supplement: awae148_Supplementary_Data [file awae148_supplementary_data.zip › brain-2023-02614-File008.pdf]

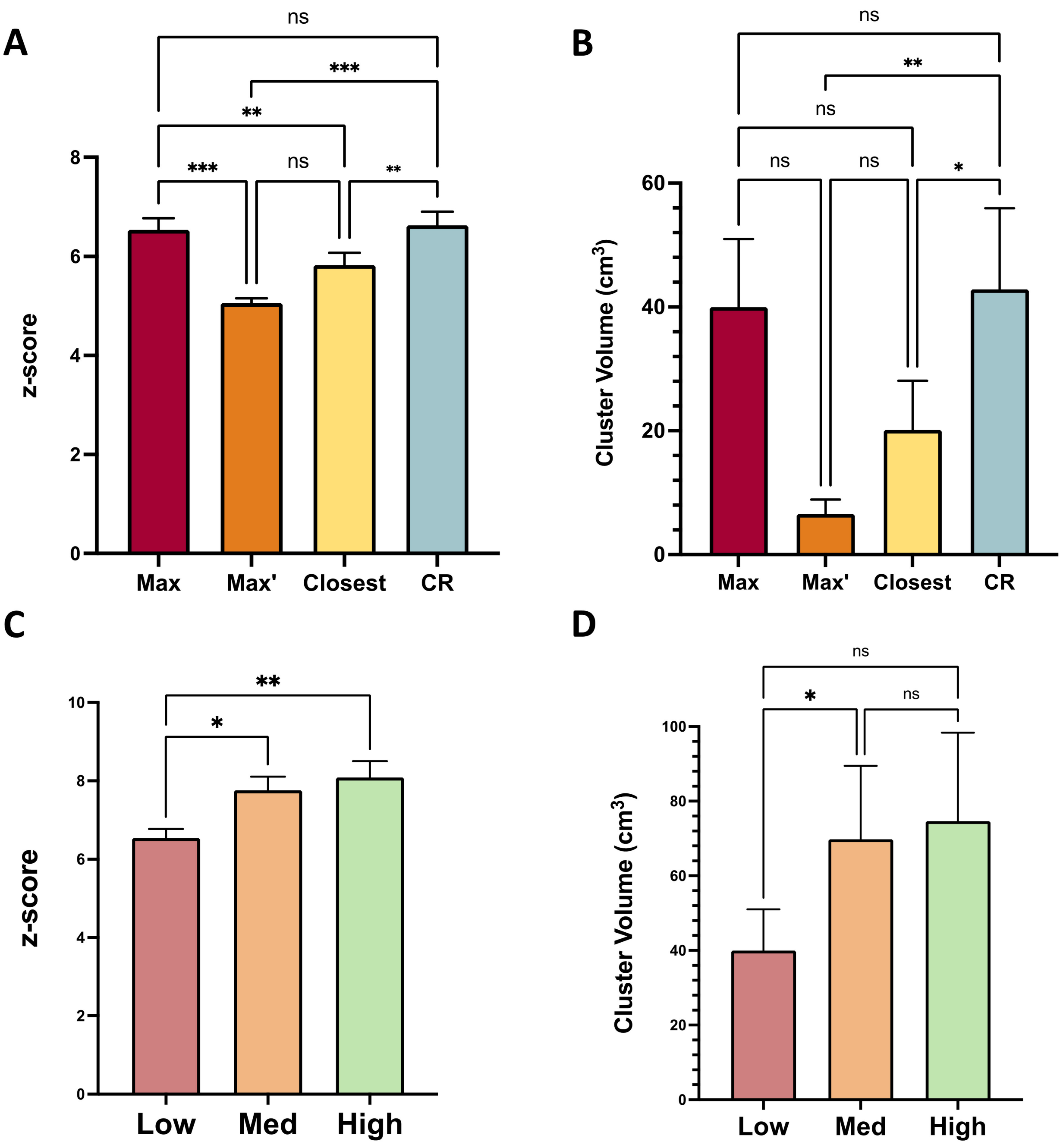

Supplementary Figure 1 Characteristics of the BOLD clusters of interest. (A) Mean z-score and **(B)** mean cluster volumes of each BOLD cluster of interest for all IED types studied. **(C)** Mean z-score and **(D)** mean cluster volume for all IED-related Maximum BOLD clusters that were classified as low, medium, or high confidence. Note that higher confidence clusters are necessarily also included in the group mean calculation for the lower confidence groups. That is, the medium confidence group is a subset of the low confidence group, and the high confidence group is a subset of both the medium and low confidence groups. Significant differences are indicated by asterisks, non-significant differences are indicated by 'ns', and error bars represent standard error.
